# Genetic Heterogeneity of Inborn Errors of Immunity Revealed by Whole-Genome Sequencing: Insights from a Russian Patient Cohort

**DOI:** 10.3390/ijms27177923

**Published:** 2026-09-05

**Authors:** Yunna Petrusenko, Tikhon Savin, Anna Sedykh, Nikolay Chekanov, Evgeny Klimuk, Yulia Ostankova, Raisa Kuznetsova, Anna Chernyshova, Anzhelika Milichkina, Areg Totolian, Konstantin Severinov

**Affiliations:** 1 Biotech Campus LLC., 117437 Moscow, Russia; ypetrusenko@biotc.ru (Y.P.);; 2 Shemyakin-Ovchinnikov Institute of Bioorganic Chemistry, Russian Academy of Sciences, 117997 Moscow, Russia; 3 Saint Petersburg Pasteur Institute, 197101 St. Petersburg, Russiashenna1@yandex.ru (Y.O.); totolian@pasteurorg.ru (A.T.); 4 Department of Immunology, Pavlov First Saint Petersburg State Medical University, 197022 St. Petersburg, Russia

**Keywords:** inborn errors of immunity (IEI), whole-genome sequencing (WGS), common variable immunodeficiency (CVID), X-linked agammaglobulinemia (XLA), immune dysregulation, rare variants, immune signaling pathways, DNA damage response

## Abstract

Identifying genetic cause(s) is a key step for management and treatment of patients with inborn errors of immunity (IEI). Here, in an observational cross-sectional genomic study, we analyzed whole-genome sequencing (WGS) data of 72 IEI patients from Saint Petersburg and Northwestern Russia: 42 patients with common variable immunodeficiency (CVID)-like phenotypes, 6 patients with clinically diagnosed X-linked agammaglobulinemia (XLA or Bruton’s disease), and 24 patients with other forms of IEI. Causative pathogenic and likely pathogenic variants in *BTK*, *CYBB*, *CHD7*, *AIRE*, *ATM*, *SBDS*, *NFKB1*, and *CTLA4* genes were identified in 14 (19%) patients. Variants of uncertain significance that could be linked to observed clinical phenotypes were detected in 6 patients. These included a *BTK* variant in a patient with Bruton’s disease, variants in *SH2D1A*, *SOCS1*, and *IKBKB* in patients with CVID, and variants in *CARD11* and *CD40LG* in patients with other forms of IEI. Additional rare variants that were mostly unique to individual patients were found in multiple IEI genes from the International Union of Immunological Societies (IUIS) Expert Committee 2024 list. In the CVID-like subcohort, pathway-level analysis of these rare variants revealed patterns associated with clinical manifestations. Taken together, our results expand the genetic characterization of an understudied regional IEI cohort, particularly of patients with CVID-like phenotypes, and identify genetic factors that are implicated in or may contribute to the disease.

## 1. Introduction

Inborn errors of immunity (IEI) represent a complex group of disorders caused by genetic defects affecting the development and function of the immune system [1,2]. Beyond susceptibility to infections, these conditions are now recognized to encompass a much broader spectrum of clinical manifestations resulting from complex disturbances in immune homeostasis. Patients with IEI frequently present with immune dysregulation, including autoimmunity, autoinflammation, lymphoproliferation, and malignancy [3,4,5]. The most recent report of the International Union of Immunological Societies (IUIS) Expert Committee (2024) lists more than 550 distinct IEI phenotypes caused by pathogenic variants in over 500 genes [2]. The list is far from complete, as molecular genetic diagnosis is established in roughly one third of IEI patients [6,7,8].

Common variable immunodeficiency (CVID) represents a major subgroup of IEI [5,9]. It is characterized primarily by hypogammaglobulinemia and impaired antibody responses, leading to recurrent infections [10]. CVID accounts for approximately 30% of reported primary immunodeficiencies [9] and has an estimated prevalence of 1:25,000 in European populations [10,11,12]. According to its clinical definition, CVID represents a highly heterogeneous condition and is often an umbrella diagnosis for patients with diverse clinical manifestations, including autoimmune disease, lymphoproliferation, enteropathy, and other complications of immune dysregulation [13,14]. Based on data from the European CVID Registry, CVID has been stratified into five clinical phenotypes: no complications, autoimmunity, polyclonal lymphocytic infiltration, enteropathy, and lymphoid malignancy [15]. The diagnostic yield of CVID genetic testing ranges from 8% to 54% [9,16,17,18,19]. This variability reflects differences in cohort compositions and study designs and may depend on patient inclusion criteria, cohort origin, median age, and the proportion of consanguineous individuals. Inclusion of relatively common variants with low penetrance, such as those in the *TNFRSF13B* gene, may also contribute to overestimation of the reported diagnostic yield [20,21,22]. Identification of pathogenic variants in known IEI genes frequently leads to reclassification of patients initially diagnosed with CVID to a defined monogenic IEI that presents with a CVID-like phenotype [9,23,24]. This underscores the need for comprehensive genomic approaches to better understand the genetic origin of the disease.

The widespread implementation of next-generation sequencing, including whole genome sequencing (WGS), has greatly accelerated the discovery of genetic causes of IEI. Multiple monogenic defects associated with CVID-like phenotypes in *NFKB1*, *CTLA4*, *IKZF1*, *PIK3CD* and other genes have been identified [25,26,27,28]. In addition to detecting single-gene defects, next-generation sequencing facilitated exploratory analyses of signaling pathways potentially involved in CVID pathogenesis. Several studies have reported enrichment of rare variants in immune-related pathways, including B-cell signaling, NF-κB activation, and apoptosis regulation [18,29,30]. While these findings offer initial insights into the broader molecular landscape of CVID, the integration of pathway-level variation with detailed clinical phenotypes has been limited, and the biological basis of phenotypic heterogeneity remains insufficiently understood. WGS enables the analysis of non-coding regions and polygenic associations [31,32], which may reveal novel candidate genes and variants contributing to disease susceptibility and expand the phenotypic spectrum of IEI.

Recent sequencing studies of CVID and mixed IEI cohorts from different countries reported a range of rare and unique genetic findings in affected patients [17,33,34]. However, genomic data on CVID from many populations, including those from Eastern Europe, remain limited. In this study, we report the results of analysis of WGS data in a cohort of IEI patients from Saint Petersburg and other parts of the Northwestern Federal District of Russia. Particular attention was given to individuals with CVID and CVID-like phenotypes. We aimed to characterize the genetic profile of these patients, identify monogenic diagnoses, and explore the distribution of rare potentially deleterious variants across IEI-related genes, signaling pathways, and clinical subgroups. Our findings provide new insights into the genetic basis of monogenic IEI and immunodeficiencies lacking an identifiable hereditary cause.

## 2. Results

### 2.1. Clinical Characteristics of the Study Cohort

A cohort of 72 patients (42 with a CVID-like phenotype and 30 with other IEI diagnoses) was recruited for the study. The main characteristics of the cohort are summarized in Table 1. Detailed information for each individual patient is provided in Supplementary Table S1. The CVID-like patients demonstrated substantial phenotypic heterogeneity according to clinical classification of non-infectious complications and were distributed across the clinical subtypes of CVID: 13 had no non-infectious complications, 12 presented with autoimmune manifestations, 12 showed features of polyclonal lymphocytic infiltration, 4 developed lymphoid malignancies, and one patient exhibited enteropathy (Supplementary Table S2). The group of IEI patients included a broad spectrum of IEI diagnoses, with X-linked agammaglobulinemia (*n* = 6) representing the most common defined condition. 12 patients were diagnosed with an unclassified immunodeficiency. Except for two brothers diagnosed with Bruton’s disease, the patients were not related to each other.

**Figure 1:**
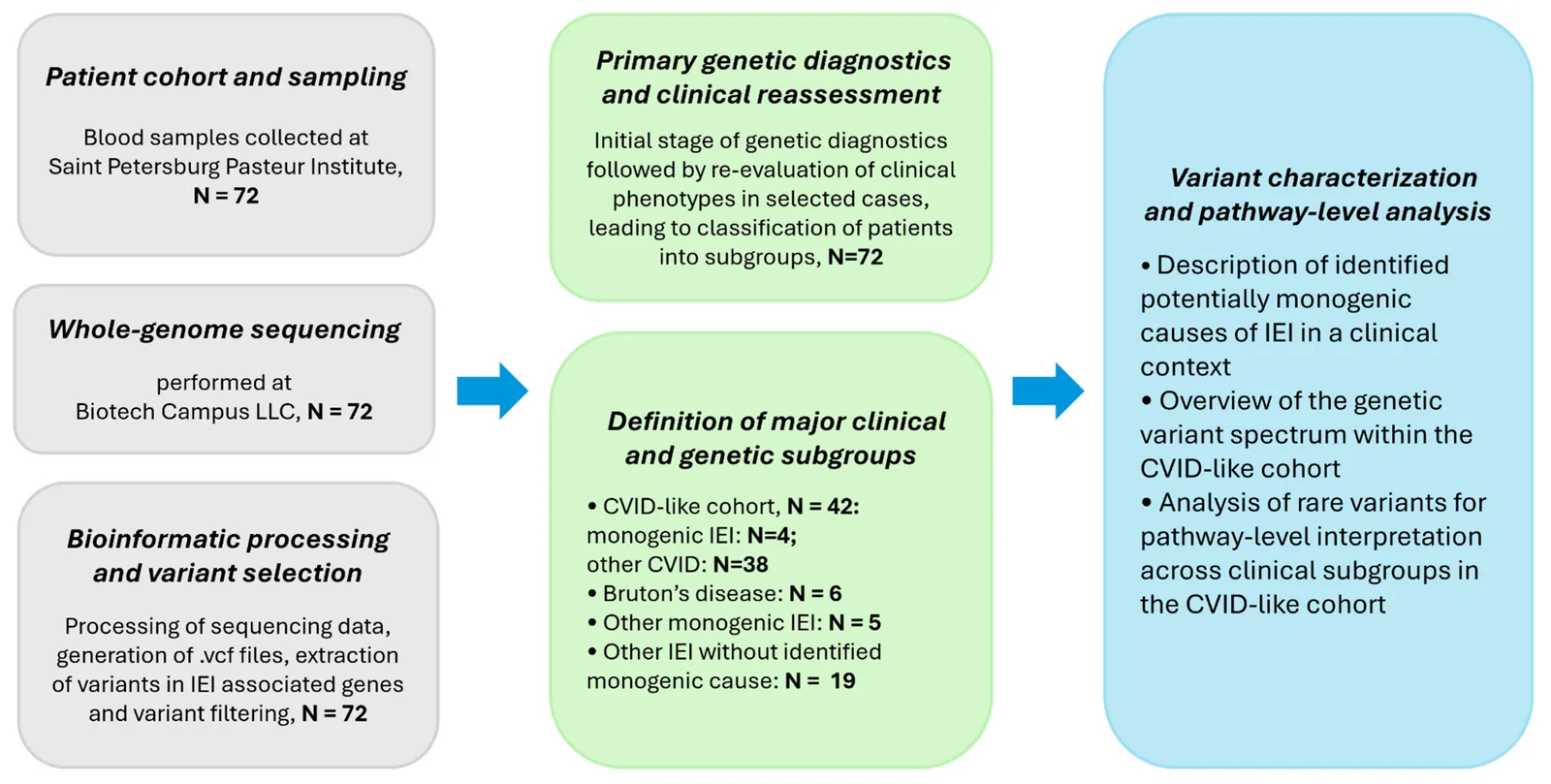
Study design and workflow. Schematic representation of the study from patient sampling and whole-genome sequencing to the genetic and clinical classification of the cohort. The subsequent analyses focused on potentially monogenic IEI and rare-variant patterns in the CVID-like cohort. IEI, inborn errors of immunity; CVID, common variable immunodeficiency; VCF, variant call format.

An overview of the study design and analytical workflow is schematically shown in Figure 1 and described in Materials and Methods. In what follows, we present the results of bioinformatic analysis and clinical interpretation of short genetic variants from WGS data of patients from our cohort.

### 2.2. Monogenic Findings in the IEI Subcohort

Pathogenic or likely pathogenic variants consistent with monogenic IEI explained the observed clinical phenotypes in 10 patients (Table 2). Among these, there were the two brothers with Bruton’s disease. Because the two brothers with Bruton’s disease were considered a single familial case, the overall diagnostic efficiency in the IEI subcohort was 9/29 (~31%), which is comparable with published research [6,7,8].

Male patient P63 carried a likely pathogenic variant in the *CYBB* gene, which encodes a subunit of the NADPH oxidase complex essential for phagocyte function [50,51]. This *CYBB* c.1016C>T (p.Pro339Leu) variant has previously been listed among variants associated with X-linked chronic granulomatous disease (CGD) [35], a primary immunodeficiency characterized by impaired microbial killing and recurrent severe infections and typically caused by defects in *CYBB* gene [52]. To our knowledge, no functional studies investigating this variant have been reported. The patient presented from the neonatal period with recurrent severe bacterial and fungal infections, lymphadenopathy, hepatosplenomegaly, Crohn’s disease, and persistent inflammatory activity. Despite immunosuppressive and antimicrobial therapy and partial clinical improvement, the family declined further invasive diagnostic procedures and hematopoietic stem cell transplantation.

Patient P8 was diagnosed with autoimmune polyendocrine syndrome type 1 (APS1 or APECED). Genetic analysis revealed a homozygous pathogenic variant in the *AIRE* gene, that is important for maintaining central immune tolerance [53]. The patient had early-onset recurrent febrile episodes with abdominal and joint symptoms, recurrent pneumonias and chronic mucocutaneous candidiasis with hepatosplenomegaly, complicated by adrenal insufficiency, hypopituitarism, autoimmune hepatitis, and thalassemia minor, with good disease control achieved on tacrolimus therapy.

Patient P30 carried two heterozygous pathogenic variants in the *ATM* gene. The patient presented with progressive gait instability beginning in early childhood, and genetic findings confirmed the clinical suspicion of ataxia–telangiectasia, a disorder characterized by neurodegeneration, immunodeficiency, and increased cancer susceptibility [54,55].

In the three cases presented above, the genetic findings confirmed the original clinical diagnoses. Patients P38 and P51 were originally diagnosed with “unclassified IEI”. Analysis of WGS data revealed specific causes of the disease.

In patient P38, a stop-gained variant in *CHD7* (rs886040991) was identified. Pathogenic variants in *CHD7* cause CHARGE syndrome, a multisystem disorder that can include immune abnormalities [39,56]. The patient had multiple congenital malformations compatible with CHARGE syndrome and recurrent bacterial infections, for which intravenous immunoglobulin replacement therapy (IVIG-RT) was initiated, leading to stabilization of his condition.

Patient P51 had two variants in the *SBDS* gene. One variant, c.258+2T>C, is a well-established pathogenic splice-site mutation frequently reported in patients with the Shwachman–Diamond syndrome (SDS) and known to arise through gene conversion events between *SBDS* and its pseudogene *SBDSP1* [41,42]. The second variant, c.410T>G (p.Met137Arg) has been previously reported in trans with c.258+2T>C in a patient with SDS [43]. Multiple tools consistently predict its deleterious effect; the variant is located in a highly conserved region with no reported benign missense substitutions and is ultra-rare in population databases. The combination of these *SBDS* variants together with the patient’s clinical presentation support the diagnosis of Shwachman–Diamond syndrome. The patient had an anorectal malformation requiring multiple surgeries, later developed type 1 diabetes mellitus with immune cytopenias and recurrent severe infections (including mandibular osteomyelitis), leading to immunologic evaluation and WGS.

**Figure 2:**
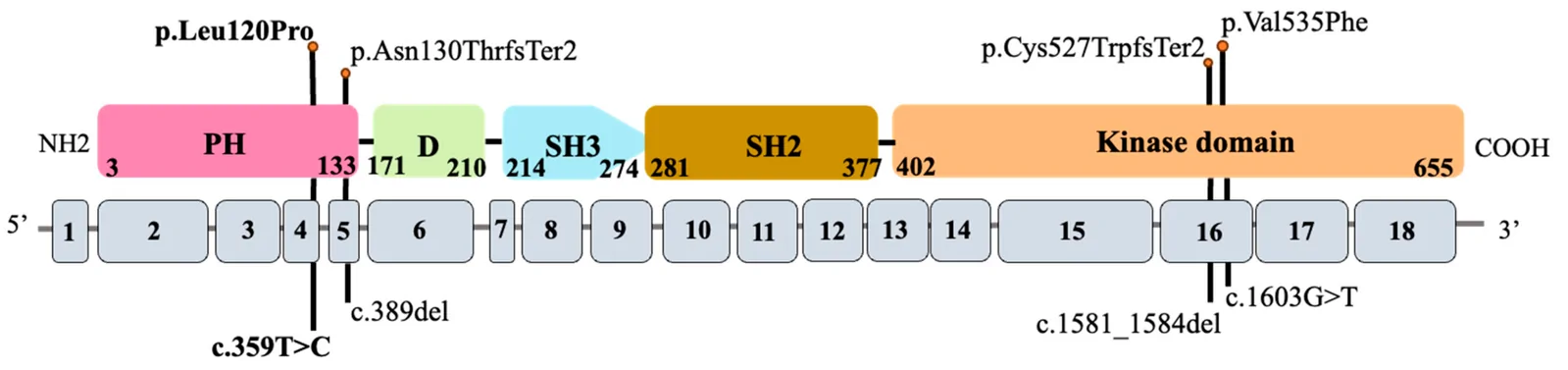
*BTK* variants identified in patients from the study cohort. Schematic representation of the domain organization of the BTK protein (Bruton’s tyrosine kinase; UniProt Q06187-1) and the structure of its gene. The protein contains five domains: PH (Pleckstrin Homology domain, aa 3–133), D (disordered region, aa 171–210), SH3 (Src Homology 3 domain, aa 214–274), SH2 (Src Homology 2 domain, aa 281–377), and the Kinase domain (aa 402–655). Four nucleotide variants are shown: c.359T>C (p.Leu120Pro) is classified as a variant of uncertain significance (highlighted in bold); c.389del (p.Asn130ThrfsTer2) within the PH domain, c.1581_1584del (p.Cys527TrpfsTer2), and c.1603G>T (p.Val535Phe) within the kinase domain are classified as likely pathogenic and associated with X-linked agammaglobulinemia (XLA) [44,46,58].

In six male patients (including the two brothers), four distinct variants in the *BTK* gene were detected (Figure 2). Pathogenic variants in this gene lead to XLA [57].

A frameshift variant c.1581_1584del (p.Cys527TrpfsTer2) was identified in three patients (P35 and the P24/P25 brothers). Family screening confirmed that the mother of P24 and P25 was a heterozygous carrier of this variant. All three patients presented with early-onset recurrent bacterial infections, including otitis media, pneumonia, and sinusitis, and were subsequently diagnosed with agammaglobulinemia. Initiation of intravenous immunoglobulin therapy led to reduction in infectious events.

Another frameshift *BTK* variant, c.389del (p.Asn130ThrfsTer2), was identified in patient P43, who also had a family history of XLA. The clinical presentation included recurrent respiratory infections beginning in infancy and a marked reduction in infectious episodes following IVIG therapy.

A missense variant c.1603G>T (p.Val535Phe), classified as likely pathogenic, was detected in patient P101. This patient had a long history of recurrent bacterial infections since early childhood, with a significant clinical improvement after initiation of immunoglobulin replacement therapy.

Finally, a missense *BTK* variant c.359T>C (p.Leu120Pro) was identified in patient P75. This variant has been previously reported in ClinVar as a variant of uncertain significance (VUS). Re-evaluation according to ACMG criteria did not provide sufficient evidence for reclassification. The variant is absent from population databases and affects a highly conserved residue within the pleckstrin homology (PH) domain of BTK, a functionally critical region required for membrane localization and signal transduction. The patient had recurrent purulent otitis media from early childhood. At the age of 4 years, agammaglobulinemia with the absence of B cells was revealed, after which IVIG therapy was initiated and effectively prevented further infections.

### 2.3. Potentially Relevant Variants of Uncertain Significance in the IEI Cohort

Variants of uncertain significance corresponding to clinical manifestations were identified in two patients, P45 and P127, initially diagnosed, respectively, with BENTA-like condition (B-cell expansion with NF-κB and T-cell anergy syndrome) and hyper-IgM syndrome (see Table 2). Full details of variants identified in patients of the subcohort are provided in Supplementary Table S3.

In patient P45, a *CARD11* variant (NM_032415.7:c.388T>G; p.Phe130Val) was detected. It affects residue Phe130 located within the LATCH domain of CARD11, a critical regulatory region where multiple gain-of-function missense variants associated with BENTA have been reported [59]. Another missense change affecting the same residue (p.Phe130Cys) has been experimentally shown to increase NF-κB activation predicting a gain-of-function effect [60]. Phe130Val has also been reported as a somatic diffuse large B-cell lymphoma-specific *CARD11* mutation and, in functional assays, was shown to increase canonical NF-κB signaling by enhancing p65 activation compared with wild-type *CARD11* [48,49]. In our case, this variant was identified as a germline change.

In patient P127, a hemizygous variant in *CD40LG* (NM_000074.3:c.716T>G; p.Val239Gly) was found. The variant is absent from population databases and is located within the TNF homology domain (THD) of CD40L, a region critical for trimerization and interaction with the CD40 receptor on B cells and antigen-presenting cells [61]. Variants in *CD40LG* cause X-linked hyper-IgM syndrome (XHIM) [62] and alterations in the THD domain may impair CD40-CD40L interaction and disrupt T-cell-dependent B-cell activation [63,64].

### 2.4. Monogenic Findings in the CVID-like Subcohort

Analysis based on genes included in the 2024 IUIS classification of inborn errors of immunity did not identify ClinVar-listed pathogenic variants explaining the phenotypes of patients in the CVID-like subcohort. The only exceptions are variants in *TNFRSF13B* that were detected in two individuals (Table 3). However, these variants are considered genetic susceptibility factors rather than definitive monogenic causes of disease due to their relatively high frequency in healthy populations and incomplete penetrance [20,65]. In four patients, we identified several likely pathogenic variants in genes known to cause monogenic immune dysregulation. These included three previously unreported variants and one variant reported in the literature but absent from major databases at the time of this writing. When considering these four variants, the diagnostic yield in the CVID-like subcohort is thus 9.5%.

Patients P4 and P74 carried heterozygous frameshift variants in the *NFKB1* gene (Table 3). Truncating variants in *NFKB1* cause CVID-like conditions through haploinsufficiency and are associated with a combination of humoral immunodeficiency, autoimmunity, and lymphoproliferation [25,69]. Clinically, both patients presented with recurrent sinopulmonary infections beginning in childhood. P4 developed bilateral bronchiectasis despite IVIG replacement therapy and later manifested splenomegaly, inflammatory bowel disease, and early-stage liver fibrosis. P74 experienced recurrent pneumonias, including severe viral–bacterial pulmonary infections in adulthood, and was found to have profound hypogammaglobulinemia with B-cell deficiency, leading to IVIG-RT initiation. He was also diagnosed with ankylosing spondyloarthritis.

Two *CTLA4* variants were identified in patients P6 and P73. Monoallelic mutations in *CTLA4* are known to cause CTLA-4 haploinsufficiency, a disorder characterized by immune dysregulation, hypogammaglobulinemia, lymphoproliferation, and lymphocytic tissue infiltration [70]. In patient P6, a previously undescribed heterozygous *CTLA4* variant (NM_005214.5:c.565dup; p.Met189AsnfsTer24) was found at the 3′ terminal region of penultimate exon 3. Although its proximity to the final exon-exon junction suggests that nonsense-mediated decay (NMD) is unlikely, this one-nucleotide insertion is predicted to cause a frameshift altering the downstream coding sequence. Consequently, the variant is expected to disrupt the CTLA-4 cytoplasmic domain encoded by exon 4 and containing conserved YVKM motif with tyrosine 201 [71]. This motif is known to be involved in CTLA-4 trafficking and signaling: interaction with AP-2 regulates receptor internalization, whereas phosphorylation of Y201 enables recruitment of SHP-2 and PI3K, thereby modulating downstream T-cell receptor signaling and regulatory T-cell function [72]. Clinically, P6 presented with recurrent respiratory infections, progressive lymphadenopathy with follicular hyperplasia on biopsy, mild neutropenia, and elevated β2-microglobulin levels. Following genetic diagnosis, initiation of abatacept therapy resulted in normalization of lymph node size.

In patient P73, a heterozygous *CTLA4* (NM_005214.5:c.160G>T; p.Ala54Ser) variant was found. The variant affects residue Ala54 located in the extracellular IgV domain of CTLA-4 that contributes to the ligand-binding interface with CD80/CD86 [73]. Another missense change affecting the same residue (p.Ala54Thr) has been reported in multiple individuals with CTLA-4 insufficiency and was shown to reduce CTLA-4 expression in functional studies [74,75,76]. A patient with immune dysregulation and cytopenias harboring the same c.160G>T substitution was described previously [66]; however, this variant is presently not listed in major databases. P73 manifested early-onset lymphadenopathy and persistent autoimmune cytopenias, including neutropenia and thrombocytopenia, AIHA with a positive direct Coombs test. Immunosuppressive therapy (GCS, CsA, MMF) provided limited benefit, whereas initiation of abatacept in combination with intravenous immunoglobulin led to sustained hematological remission and clinical stabilization.

### 2.5. Potentially Relevant Variants of Uncertain Significance in the CVID-like Cohort

Three rare variants of uncertain significance were identified in *SH2D1A, SOCS1*, and *IKBKB* genes involved in immune regulation (Table 3). Although currently lacking definitive functional confirmation, these variants affect evolutionarily conserved residues and/or critical functional domains and therefore may contribute to immune dysregulation in patients with CVID-like phenotypes.

A frameshift variant in *SH2D1A* (NM_002351.5:c.269dup; p.Asp91ArgfsTer13) was identified in a 44-year-old male patient P84 diagnosed with CVID. *SH2D1A* encodes the SLAM-associated protein (SAP), an adaptor that mediates signaling through SLAM family receptors and is mainly expressed in T cells and NK cells [77,78]. Loss-of-function variants in *SH2D1A* cause X-linked lymphoproliferative syndrome type 1 (XLP1) [79,80]. The detected variant is located in exon 3 within the SH2 domain of SAP and introduces a premature stop codon. The gene is known to be intolerant to loss-of-function variation, and multiple pathogenic mutations have been reported in nearby positions [80,81]. Whole-genome sequencing did not identify additional variants in *SH2D1A* that could compensate for or modify the frameshift event. In the absence of functional validation the variant was classified as a VUS. Clinically, the patient had a history of recurrent maxillary sinusitis beginning at 14 years of age. In 2012, he developed severe infectious mononucleosis requiring intensive care unit admission, accompanied by prolonged Epstein–Barr virus (EBV) viremia. Thereafter, morbidity of infections increased, with recurrent sinusitis and pneumonias. In 2019, immunological evaluation established a diagnosis of CVID with a predominant impairment of B-cell number and function; persistent EBV viremia was also documented. Since 2019, he has been receiving continuous IVIG replacement therapy. Despite the predicted loss-of-function effect and the clinical history of severe EBV infection frequently observed in SH2D1A deficiency [81], the patient survived into adulthood without hemophagocytic lymphohistiocytosis or overt lymphoproliferative disease typical for classical XLP1 [82]. Due to this discrepancy between the predicted molecular effect and the clinical presentation, we downgraded the ACMG PVS1 criterion to moderate.

**Figure 3:**
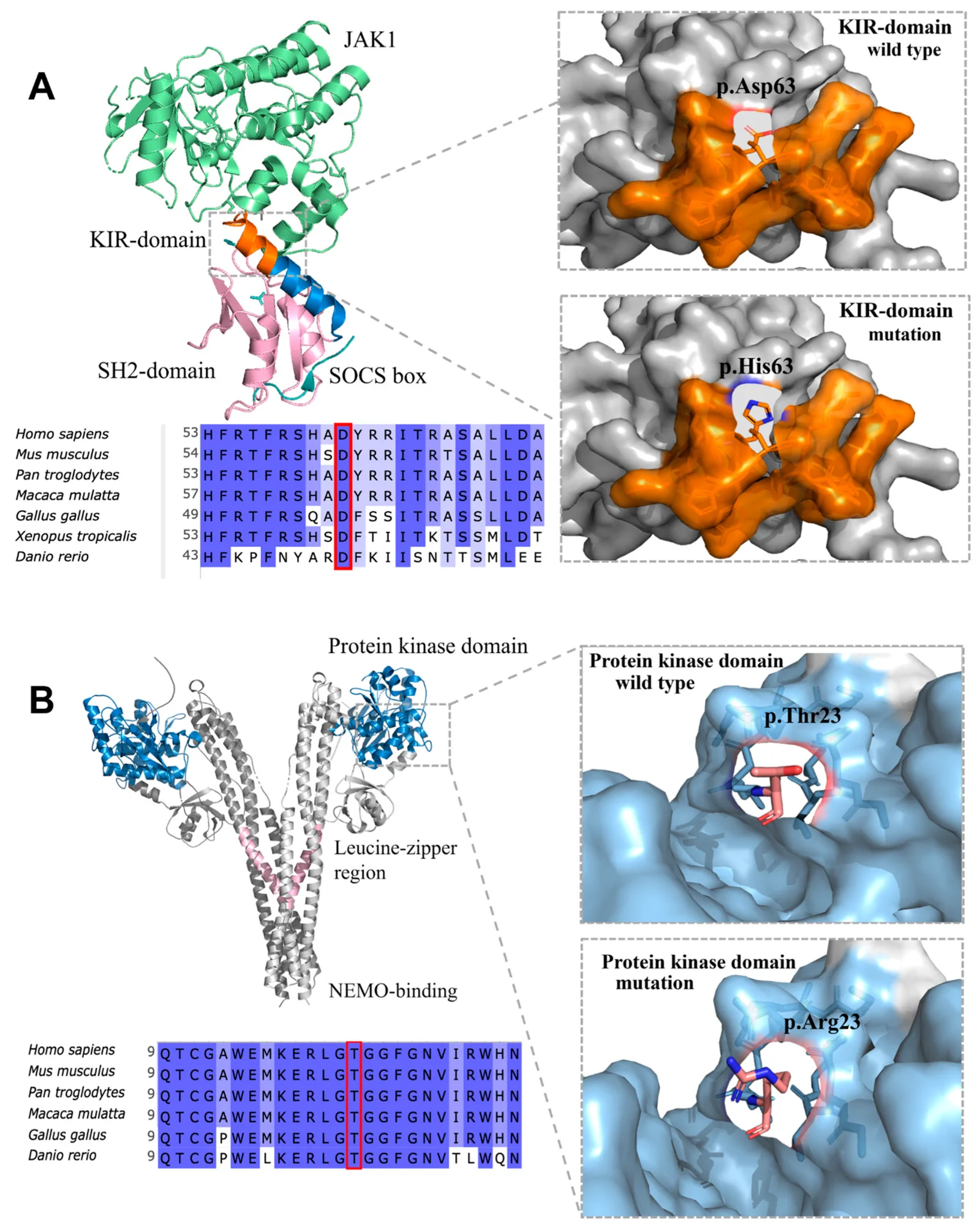
Structural context and evolutionary conservation of identified SOCS1 and IKBKB variants. (**A**) The structure of the SOCS1 protein showing its functional domains. The Asp63 residue substituted to His in patient P13 is located within the KIR domain, a critical region responsible for direct inhibition of JAK kinase activity. Multiple sequence alignment of SOCS1 orthologs across representative vertebrate species demonstrating conservation of the KIR containing Asp63 is shown below. (**B**) Structural organization of the IKBKB protein and localization of the Thr23Arg substitution. On the structure, the protein kinase domain, leucine zipper region, and NEMO-binding domain are indicated. The Thr23Arg substitution found in patient P172 is located within the kinase domain. Multiple sequence alignment of IKBKB orthologs across representative species is presented below. The species included in the multiple-sequence alignments are *Homo sapiens* (human), *Mus musculus* (mouse), *Pan troglodytes* (chimpanzee), *Macaca mulatta* (rhesus macaque), *Gallus gallus* (chicken), *Xenopus tropicalis* (Western clawed frog), and *Danio rerio* (zebrafish). Red boxes highlight the alignment positions affected by variants identified in the patients.

A heterozygous missense variant in *SOCS1* (NM_003745.2:c.187G>C; p.Asp63His) was identified in a 38-year-old female (P13). SOCS1 is a critical negative regulator of the JAK–STAT signaling pathway and plays a central role in controlling cytokine responses, particularly those mediated by interferon-γ [83]. The identified variant affects Asp63 within the kinase inhibitory region (KIR), which directly suppresses JAK kinase activity [84]. This residue is highly conserved across vertebrates, supporting its functional importance (Figure 3A); while in silico structural assessment revealed no evidence of global structural disruption (Supplementary Figure S1A). The patient remained clinically asymptomatic until 2015, when she began to experience recurrent episodes of pneumonia necessitating hospitalization. In 2018, common variable immunodeficiency with predominant impairment of B-cell number and function was diagnosed, and IVIG-RT therapy was initiated. The disease course was complicated by lymphoid interstitial pneumonia with granulomatous changes confirmed by lung biopsy and progressive interstitial abnormalities on chest CT. Due to significant adverse reactions to IVIG and progressive pulmonary involvement, abatacept therapy was initiated in 2025 and was well tolerated.

A heterozygous missense variant in *IKBKB* (NM_001556.3:c.68C>G; p.Thr23Arg) was identified in a 45-year-old male (P172). *IKBKB* encodes IKKβ, a catalytic component of the IκB kinase complex required for activation of NF-κB signaling [85]. The p.Thr23Arg substitution affects a conserved residue within the ATP-binding region of the kinase domain [86] and is predicted to interfere with kinase activity (Figure 3B), without evidence of major global structural disruption in the mutant model (Supplementary Figure S1B). The patient had recurrent upper respiratory tract infections since childhood and, from early adulthood, frequent purulent sinusitis, tonsillitis, otitis media, and recurrent herpes labialis. Immunological evaluation in 2023 revealed marked hypogammaglobulinemia with reduced IgG subclasses. CVID was diagnosed in 2023, and IVIG-RT was initiated in 2024, resulting in clinical stabilization. No systemic autoinflammation or ectodermal abnormalities were observed. In the absence of functional validation, the variant was classified as a VUS.

In addition to three variants discussed above, several non-coding VUSs were detected in genes associated with CVID-like conditions in our subcohort. The annotation and genomic coordinates of these variants are provided in the Supplementary Table S4. As can be seen, these variants are located within putative regulatory elements predicted by ENCODE and thus might contribute to patients’ conditions; however, this requires further functional analysis.

### 2.6. Gene-Level Distribution of Rare Variants in the CVID Subcohort

To explore the broader genetic landscape of the CVID subcohort, we examined the distribution of rare variants across genes included in the IUIS inborn errors of immunity gene list, a curated classification of genes associated with monogenic immune disorders established by the International Union of Immunological Societies [2]. This list also includes genes involved in DNA repair and cancer predisposition (e.g., *BRCA2*, *PALB2*, *ATM*, *NBN*, *MSH6*, and *BRIP1*) [87], which were retained in the analysis due to their presence in the diagnostic panel and their potential relevance to immune dysregulation and malignancy in CVID-like phenotypes [2].

**Figure 4:**
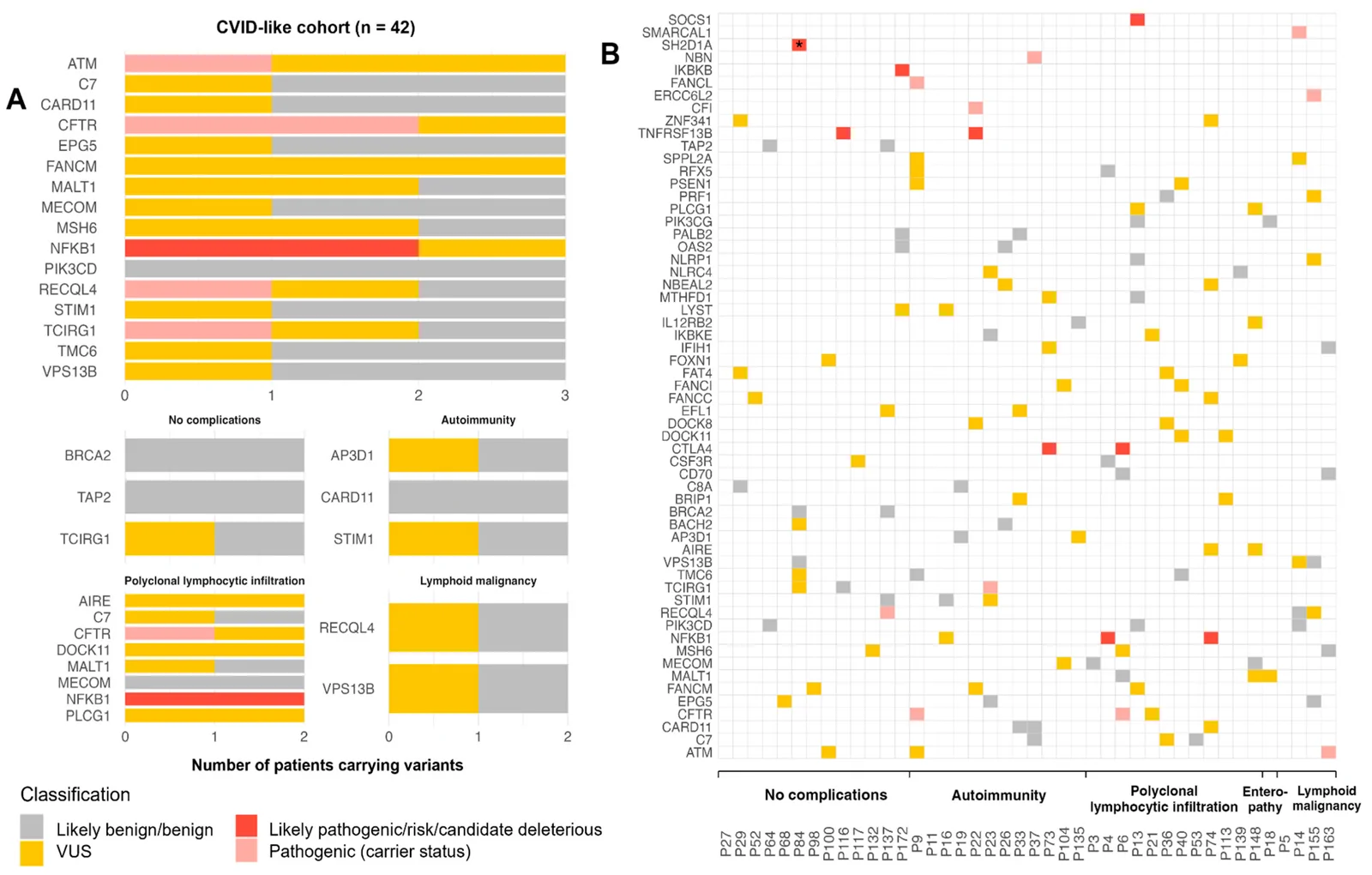
The distribution of rare variants across the CVID-like cohort. (**A**) Genes from the IUIS inborn errors of immunity gene list recurrently affected by rare variants. Top: distribution across the entire CVID-like cohort (*n* = 42). Only genes with variants identified in ≥2 patients are shown. Bottom: distribution across the clinical subgroups: no complications (*n* = 13), autoimmunity (*n* = 12), polyclonal lymphocytic infiltration (*n* = 12), and lymphoid malignancy (*n* = 4). The enteropathy subgroup was excluded from this analysis due to insufficient size (*n* = 1). (**B**) Tile plot summarizing variants’ occurrence across the entire CVID-like cohort. Each tile indicates the presence of at least one rare variant in an indicated gene for a given patient (see Materials and Methods for filtering criteria). Tile color denotes variant combined classification (likely benign, VUS, or likely pathogenic/risk/candidate deleterious). An asterisk indicates a variant present in the homozygous or hemizygous state. A total of 59 IEI associated genes from the IUIS gene list are shown, selected based on the presence of at least one likely pathogenic/risk/candidate deleterious variant or more than one variant classified as likely benign or VUS in the cohort.

Rare variants (after filtering described in Materials and Methods) were detected across a wide range of genes from the list, with no single gene being recurrently affected in more than 7% of patients (Figure 4A). A similar pattern was observed when patients were stratified by clinical subgroups (individuals without complications (*n* = 13), autoimmunity (*n* = 12), polyclonal lymphocytic infiltration (*n* = 12), and lymphoid malignancy (*n* = 4)): variants were distributed across different genes rather than clustering in specific loci (Figure 4).

We also attempted to group genes with rare variants observed in CVID-like patients into IEI categories, including combined immunodeficiencies, defects of innate immunity, and immune dysregulation (Supplementary Figure S2). However, this patient-normalized analysis did not reveal category-specific enrichment that could inform the underlying disease mechanisms.

### 2.7. Pathway-Level Analysis of Rare Variants in CVID Clinical Subgroups

**Figure 5:**
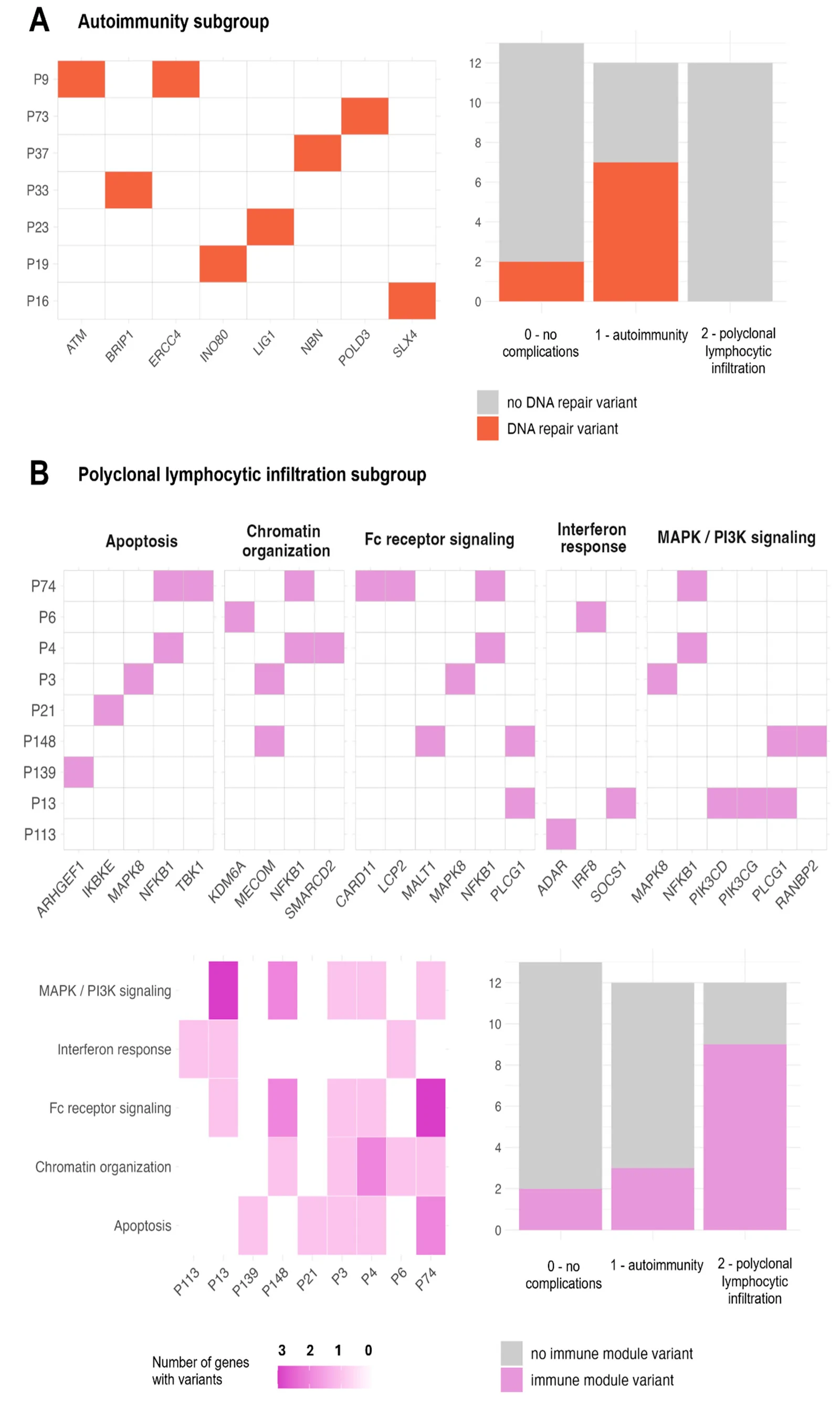
(**A**) DNA repair pathway clusters associated with autoimmune manifestations in the CVID-like group. (**Left**) A heatmap showing the distribution of rare variants in genes belonging to DNA repair pathway clusters among patients with autoimmune manifestations. Columns represent genes and rows represent individual patients; colored cells indicate the presence of a variant. (**Right**) A bar plot showing the number of patients carrying variants in DNA repair pathway genes across the three clinical CVID subgroups. (**B**) Immune signaling modules associated with polyclonal lymphocytic infiltration in the CVID-like group. (**Top**) A heatmap showing the distribution of rare variants across genes belonging to enriched immune signaling pathways in patients with lymphoproliferation. Genes are grouped into functional immune modules derived from pathway clustering. (**Bottom left**) Module-level heatmap summarizing variant burden per patient; columns represent patients and rows represent immune modules, with color intensity indicating the number of genes with variants per module (0–3). (**Bottom right**) A bar plot showing the number of patients carrying variants in genes within the identified immune signaling modules across the three clinical CVID subgroups.

To investigate whether rare variants detected in patients with CVID converge on specific biological processes, we examined signaling pathways of which the IEI genes carrying rare variants with high predicted pathogenicity (CADD ≥ 20) were part of. This approach allows one to detect shared biological processes potentially affected by variants occurring in different genes across patients. Genes harboring such variants were mapped to KEGG and Reactome pathways and overlapping pathways were collapsed into clusters based on gene overlap to reduce redundancy (Supplementary Figure S3). The overall distribution of pathway-level signals across clinical subgroups is summarized in Supplementary Figure S4 and gene-level variant patterns within the main pathway clusters are shown in Figure 5. In the subgroup of patients with autoimmune complications (*n* = 12), nominal enrichment trends (*p* < 0.05) were observed for pathways related to DNA damage response, particularly homologous recombination and nucleotide excision repair pathways (Reactome). 7 out of 12 patients in this subgroup carried rare variants with high predicted pathogenicity in genes involved in DNA repair. Examination of gene-level contributions within these pathways indicated that multiple genes contributed to the observed signal rather than a single recurrent gene effect (Figure 5A). While none of the pathways remained significant after multiple testing correction (FDR < 0.05)—likely due to the limited cohort size—the observed nominal enrichment of DNA repair pathways suggests biologically coherent trends that warrant further investigation in larger independent cohorts.

In patients with polyclonal lymphocytic infiltration (*n* = 12), enrichment trends were observed for pathways associated with immune signaling and cellular activation. These pathways form several related functional modules, including Fc receptor signaling, MAPK/PI3K signaling cascades, interferon signaling, apoptosis, and chromatin organization (Figure 5B). 9 of 12 patients in this subgroup carried rare variants in genes belonging to pathways showing nominal enrichment (*p* < 0.05). In several cases, individual patients harbored variants in multiple genes within related immune signaling pathways. However, none of the pathways remained significant after correction for multiple testing (FDR < 0.05). Complete Fisher’s exact test results and the corresponding pathway-level enrichment outputs for each clinical subgroup are provided in Supplementary Table S5–S8.

## 3. Discussion

Below, we discuss the key genomic findings in our cohort and their diagnostic implications across the spectrum of IEI phenotypes.

Several patients in the cohort were found to carry pathogenic variants consistent with well-established monogenic disorders associated with immune dysfunction. These included variants in *CYBB*, *AIRE*, *CHD7*, *SBDS*, and *ATM*, corresponding to chronic granulomatous disease, autoimmune polyendocrine syndrome type 1, CHARGE syndrome, Shwachman-Diamond syndrome, and ataxia-telangiectasia, respectively. In these cases, the genetic findings showed a clear genotype-phenotype correlation and either confirmed or clarified the clinical diagnosis. Such results highlight the diagnostic value of comprehensive genomic analysis in patients with suspected inborn errors of immunity, particularly when clinical manifestations overlap with syndromic or multisystem disorders.

Mutations in the *BTK* gene represented the most frequent genetic cause identified in this cohort, with six patients, which included five who were unrelated to each other, carrying likely pathogenic variants or VUS consistent with XLA. Both truncating and missense variants were observed, including a recurrent frameshift mutation, supporting the role of familial screening in X-linked disorders.

A subset of patients initially classified as having CVID-like immunodeficiency carried variants consistent with monogenic immune dysregulation disorders. In particular, likely pathogenic variants in *NFKB1* and *CTLA4* were identified, both of which are well-recognized genetic causes of CVID-like phenotypes. These findings support the growing evidence that clinically defined CVID represents a genetically heterogeneous group of disorders. The relatively modest diagnostic yield observed in our cohort (9.5%) may be related to the later age of onset and higher median age, factors that are generally associated with a lower likelihood of identifying highly penetrant monogenic causes, which are more commonly observed in early-onset severe diseases [18,88].

Beyond variants classified as pathogenic, our analysis also identified several rare variants of uncertain significance in genes involved in immune signaling pathways, including *SH2D1A*, *SOCS1*, and *IKBKB*. Although the pathogenicity of these variants cannot be confirmed, their location within conserved residues or key functional domains suggests potential biological relevance.

The *SH2D1A* frameshift variant identified in one patient is predicted to truncate the SAP adaptor protein that mediates SLAM-dependent signaling in T and NK cells. While classical SH2D1A deficiency causes X-linked lymphoproliferative syndrome [82], the relatively mild clinical course observed in this patient suggests phenotypic variability associated with possible disruption of this pathway [89,90,91].

The *SOCS1* p.Asp63His substitution affects a conserved residue within the kinase inhibitory region responsible for suppressing JAK–STAT signaling. Although *SOCS1* haploinsufficiency is typically associated with early-onset systemic autoimmunity and autoinflammatory manifestations [92], the phenotype observed in our patient is dominated by immunodeficiency and lymphoproliferative lung disease. Similar CVID-like phenotypes have only rarely been reported in association with *SOCS1* variants [31,93].

Finally, the *IKBKB* p.Thr23Arg variant affects a conserved residue within the ATP-binding region of the IKKβ kinase domain. While pathogenic *IKBKB* variants are typically associated with either combined immunodeficiency or inflammatory syndromes [94,95,96], the clinical phenotype in this patient resembled CVID with predominant antibody deficiency. This may reflect a partial impairment of NF-κB signaling, as heterozygous *IKBKB* variants have also been described in some patients with a CVID-like phenotype [29,97]. The functional impact of this variant requires experimental validation.

Gene-level analysis demonstrated considerable genetic diversity within the cohort. Rare variants were distributed across numerous IEI genes, and no single gene was recurrently affected in a large proportion of patients, even within clinically defined subgroups. This pattern is consistent with the well-recognized genetic complexity of CVID, suggesting that additional factors or more complex genetic architectures beyond single-gene variants may contribute to disease development [98,99].

We explored whether rare variants detected in patients with CVID converge on particular biological pathways associated with specific clinical manifestations. While no pathway clusters reached statistical significance after correction for multiple testing (FDR < 0.05), likely reflecting the limited cohort size, several nominally enriched pathways converged on shared biological processes within the clinical subgroups, and functionally related modules tended to appear together in the analysis.

In patients with autoimmune manifestations, we observed nominal enrichment of DNA damage response (DDR) pathways, including nucleotide excision repair and homology-directed repair modules. Rare variants were identified in several DDR-related genes (*ATM*, *BRIP1*, *ERCC4*, *NBN*, *POLD3*, and *SLX4*), suggesting involvement of complementary genome maintenance mechanisms. These findings align with prior reports of impaired ATM signaling in subsets of CVID patients, characterized by reduced irradiation-induced ATM phosphorylation and altered 53BP1 and γH2AX dynamics, as well as dysregulated expression of DNA repair genes such as increased ERCC2 and reduced MSH6 and TREX1 [100] and support the concept of disturbed genome maintenance in complicated CVID phenotypes.

The lymphoproliferative subgroup demonstrated coordinated enrichment of the immune receptor–proximal and downstream signaling modules, including Fc receptor signaling, MAPK/PI3K pathways, interferon response, chromatin organization, and death receptor signaling (Figure 5B). Several patients within our lymphoproliferative subgroup carried rare variants in multiple genes belonging to related immune signaling modules, suggesting that perturbations affecting different components of the same signaling networks may coexist within individual patients. These results partially converge with previous genomic studies of CVID. For example, whole-genome and RNA sequencing analyses have reported enrichment of B-cell receptor and apoptosis-related pathways [29], while pathway-burden analyses in a Spanish cohort identified excess rare functional variation in B-cell signaling, tumor necrosis factor, and Fc epsilon RI pathways [18]. Overall, these findings further illustrate the marked genetic and clinical heterogeneity of CVID and highlight the potential value of integrative pathway-based approaches for investigating complex immunodeficiency phenotypes. A distinguishing feature of our analysis is the implementation of detailed clinical stratification. However, the inferences drawn here require validation in larger cohorts. In aggregate, pathway-level analysis might reveal biologically meaningful trends that are not readily detectable when considering individual genes in isolation.

Several additional limitations of the study should be acknowledged. First, the genetic analysis was restricted to genes included in the IEI panel and did not capture potentially relevant variants in genes not currently associated with IEI. Second, a formal genome-wide copy number variation (CNV) analysis was not systematically performed for all patients, and smaller copy number variants may have remained undetected. Third, the relatively small cohort size limits statistical power and requires cautious interpretation of the observed trends, particularly in the pathway enrichment analysis. Finally, functional validation of the identified variants was beyond the scope of our study, and thus their biological impact remains to be established. Comprehensive in silico approaches integrating multiple prediction algorithms, structural modeling, and evolutionary conservation analysis provide valuable evidence for variant prioritization prior to functional testing. Such an approach has recently been used to evaluate variants associated with hereditary angioedema [101]. Extending this framework to VUSs identified in our cohort could provide additional lines of evidence to support or refute their causal role in CVID-like phenotypes.

Despite these limitations, our results emphasize the value of genomic approaches for improving the molecular diagnosis of inborn errors of immunity. Future studies combining genome-wide sequencing approaches, comprehensive CNV analysis, larger patient cohorts, and functional assays will be essential to further clarify the contribution of rare variants and pathway-level perturbations to the pathogenesis of CVID.

## 4. Materials and Methods

### 4.1. Study Cohort and Clinical Classification

This was a single-center observational cross-sectional study with genotype–phenotype analyses of patients with inborn errors of immunity evaluated at the Medical Center of the Saint Petersburg Pasteur Institute, which provides specialized care to patients from the Northwestern Federal District of Russia. While we did not investigate the ethnic background of the patients by means of genetics, they all identified themselves as Russians. Patients with a definite or probable diagnosis of IEI and no history of hematopoietic stem cell transplantation (HSCT) were eligible for inclusion. No a priori sample-size calculation was performed and all eligible patients available during the study period were included.

Clinical and immunological data were obtained during routine diagnostic evaluation and clinical follow-up at the Medical Center of the Saint Petersburg Pasteur Institute. Baseline clinical and immunological assessments, including lymphocyte subset phenotyping and serum immunoglobulin levels, were obtained in 2022–2023. Blood samples for whole-genome sequencing were collected in 2023–2024 within a research project involving patients with IEI approved by the Local Ethics Committee of the Saint Petersburg Pasteur Institute in 2023 (Protocol No. 86). The continuation of the project and analysis of collected clinical and genomic data were approved by the same Local Ethics Committee on 25 February 2025 (Protocol No. 103). Written informed consents were obtained from all participants or their legal representatives.

Diagnoses of inborn errors of immunity, including common variable immunodeficiency and unclassified immunodeficiencies, were established in accordance with the ESID Registry Working Definitions for Clinical Diagnosis of IEI [9]. CVID clinical subtypes were classified according to Chapel et al. [15] Although the Chapel et al. classification recognizes overlapping clinical phenotypes, for the purposes of the present study, each patient was assigned to a single clinical subgroup based on the dominant clinical manifestation. The diagnostic categorization of monogenic IEI was based on the identified genetic variants. The study was reported in accordance with the Strengthening the Reporting of Observational Studies in Epidemiology (STROBE) guidelines.

### 4.2. Sequencing and Bioinformatics Analysis

Sequencing and primary data processing were performed at Biotech Campus LLC. Whole-genome sequencing was performed on a DNBSEQ-T7 platform (MGI, Shenzhen, China) using 150 bp paired-end reads, with all samples achieving a median sequencing coverage of at least 30×. Reads were aligned to the GRCh38 reference genome using BWA v0.7.17 [102]. Short variants were called using DeepVariant v1.4 [103], and a cohort-level multisample VCF file was generated using bcftools v1.21 [104]. Genotype-level quality control excluded variant calls with a read depth < 10 or genotype quality < 20. Variants were annotated using Ensembl Variant Effect Predictor v115.1 (VEP) [105], including predicted functional consequences, population allele frequencies from gnomAD v4.1 [106], and selected in silico pathogenicity prediction scores. Additional details are provided in the previously published protocol [107], which used the same sequencing technology and primary bioinformatic pipeline.

### 4.3. Variant Prioritization in Genes Associated with Inborn Errors of Immunity

Variant analysis was performed using a predefined panel of genes associated with inborn errors of immunity (IEI) according to the IUIS classification [2]. Rare variants were selected using population frequency filters based on gnomAD: gnomAD_AF_joint < 0.005 and gnomAD_MaxAF < 0.005. Additional filtering criteria included nhomalt_joint < 2 and nhomalt_grpmax < 2 to exclude variants with multiple homozygous carriers in population datasets. Here gnomAD_AF_joint indicates the total allele frequency in gnomAD; gnomAD_MaxAF indicates the highest allele frequency observed across ancestry groups; nhomalt_joint and nhomalt_grpmax metrics represent the number of homozygous alternate individuals in gnomAD overall and in the most enriched ancestry group.

Coding variants and variants affecting splice regions were considered. Variants predicted to alter splicing were prioritized based on splice annotations and high scores in splice prediction tools, including SpliceAI v1.3.1 [108]. In addition, non-coding variants located near IEI genes (e.g., upstream regions or 5′UTRs) were evaluated for potential regulatory impact in the CVID subcohort, including overlap with ENCODE candidate cis-regulatory elements (cCREs) [109].

Variants identified in IEI genes were interpreted according to ACMG guidelines [110]. Known pathogenic/likely pathogenic variants were retained even if exceeding the mentioned frequency thresholds when compatible with the patient phenotype and inheritance model.

### 4.4. In Silico Structural Prediction and Conservation Analysis

Amino acid sequences used for multiple sequence alignment were retrieved from the UniProt database [111]. Multiple sequence alignment was performed using the UGENE v53.1 software package [112] implementing the Clustal Omega v1.2.4 (ClustalO) algorithm with default parameters [113]. Three-dimensional (3D) protein structures were obtained from the Protein Data Bank (PDB) [114].

Structural visualization and analysis of the spatial localization of amino acid substitutions were conducted using PyMOL v3.1 [115]. Structural quality assessment and evaluation of mutation-induced conformational changes were performed using the MolProbity v4.5.1 server [116], which analyzes protein geometry, including Ramachandran plot statistics and steric clashes. The impact of amino acid substitutions on protein stability was predicted using the DynaMut2 web server (accessed March 2026) [117], which estimates changes in folding free energy (ΔΔG) and evaluates the potential effects of mutations on protein dynamics.

### 4.5. Rare Variant Selection for Pathway-Based Analysis

Rare variants with predicted functional impact were further analyzed at the pathway level. Given the relatively small cohort size, the pathway analysis was designed as an exploratory approach to identify potential biological processes associated with different clinical manifestations of CVID. Variants with a Combined Annotation Dependent Depletion (CADD) score ≥ 20 were retained, while frameshift variants were included regardless of CADD score due to their expected high functional impact. Genes harboring such variants were used for downstream pathway analysis.

### 4.6. Pathway Annotation and Network-Based Pathway Clustering

Genes containing rare variants were mapped to biological pathways using KEGG and Reactome gene sets from MSigDB v2026.1.Hs [118,119] via the R package msigdbr v26.1.0. To reduce redundancy between pathways, overlapping pathways were collapsed based on gene content similarity. Pairwise pathway similarity was calculated using the Jaccard index, and pathways with similarity greater than 0.15 were grouped using Louvain community detection to identify clusters of functionally related pathways. For each cluster, the pathway with the lowest enrichment *p*-value was selected as the representative pathway. Multiple testing correction across pathway clusters was performed using the Benjamini–Hochberg method.

### 4.7. Pathway-Level Rare Variant Burden Analysis

This analysis included 37 patients with common variable immunodeficiency (CVID). Patients were stratified into three clinical subgroups with comparable sizes based on major non-infectious complications: autoimmune manifestations (*n* = 12), polyclonal lymphocytic infiltration (*n* = 12), and patients without immune complications (*n* = 13). Patients with enteropathy (*n* = 1) and lymphoid malignancy (*n* = 4) were excluded from this comparison due to the small subgroup sizes.

For each pathway, patients were classified according to the presence or absence of rare variants in genes belonging to that pathway. The number of variant carriers was then compared between clinical subgroups using Fisher’s exact test to identify pathways showing subgroup-specific enrichment of rare variants.

### 4.8. Statistical Analysis

Statistical analyses were performed in the R statistical environment. Rare variants were aggregated at the pathway level. For each patient and each pathway, the presence of at least one rare variant in any gene belonging to that pathway was recorded. Each patient-pathway combination was counted only once; if multiple variants or multiple genes from the same pathway were present in a patient, they were considered as a single pathway hit.

For each pathway, enrichment of variant carriers was assessed using Fisher’s exact test. Comparisons were performed separately for each clinical subgroup by comparing patients from that subgroup with all remaining patients combined (e.g., the “autoimmunity” subgroup versus the other clinical groups). For every comparison, a 2 × 2 contingency table was constructed based on the number of patients with at least one variant in genes belonging to the pathway and those without such variants. To account for multiple testing across pathways, *p*-values were adjusted using the Benjamini–Hochberg procedure, and the resulting false discovery rate (FDR) values were reported. Data processing and visualization were performed using standard packages in R v.4.4.2.

## 5. Conclusions

This study reports whole-genome sequencing results for a regional Russian cohort with inborn errors of immunity, including adult patients with CVID/CVID-like presentations and other IEI phenotypes, thereby addressing a gap in population-specific data. Pathogenic variants underlying several monogenic immune disorders were identified and, in some cases, helped to clarify the clinical diagnosis. Rare variants of uncertain significance were also detected in genes involved in immune regulatory pathways, illustrating the challenges of variant interpretation in patients with IEI, particularly those presenting with CVID-like phenotypes.

## Figures and Tables

**Table 1:** Demographic and clinical characteristics of the cohort subgroups.

Metrics	CVID/CVID-like, *N* = 42	Other IEI Cohort, *N* = 30
**Sex: *n***	male: 18 female: 24	male: 15 female: 15
**Median age, range (min–max), interquartile range**	38.5, range 7–80, IQR 26	16, range 3–66, IQR 13
**Clinical subtype: *n***	no complications: 13 autoimmunity: 12 polyclonal lymphocytic infiltration: 12 enteropathy: 1 lymphoid malignancy: 4	XLA: 6 APECED: 1 AT: 1 CHARGE syndrome: 1 Shwachman–Diamond syndrome: 1 X-CGD: 1 BENTA possible: 1 HIgM: 1 SIgAD: 3 Unclassified disorder of immune dysregulation: 1 Unclassified antibody deficiency: 1 Unclassified immunodeficiency: 12
**Age of onset (years): *n***	<1: 1 1–5: 2 5–10: 12 10–20: 14 20–30: 7 30–40: 6	<1: 11 1–5: 10 5–10: 4 10–20: 2 20–30: 1 30–40: 1 40–50: 1

Note: *N* indicates the total number of patients in each cohort subgroup; *n* indicates the number of patients with the respective characteristic. CVID, common variable immunodeficiency; IEI, inborn errors of immunity; XLA, X-linked agammaglobulinemia; APECED, autoimmune polyendocrinopathy-candidiasis-ectodermal dystrophy; AT, ataxia-telangiectasia; CHARGE syndrome, coloboma, heart defects, atresia of the choanae, growth retardation, genital anomalies, and ear abnormalities syndrome; X-CGD, X-linked chronic granulomatous disease; BENTA, B-cell expansion with NF-κB and T-cell anergy syndrome; HIgM, hyper-IgM syndrome; SIgAD, selective immunoglobulin A deficiency.

**Table 2:** Pathogenic and candidate variants in genes associated with IEI in the IEI subcohort.

Patient	Diagnosis	Gene	Genotype	ACMG Classification	HGVS Transcript	HGVS Protein	gnomAD AF Total	ClinVar Classification (Submissions)	References
P63	X-CGD	*CYBB*	Hemi	likely pathogenic	NM_000397.4:c.1016C>T	p.Pro339Leu	-	-	[35]
P8	APECED	*AIRE*	Hom	pathogenic	NM_000383.4:c.607C>T	p.Arg203Ter	0.000008682	Pathogenic/Likely pathogenic (4)	[36,37]
P30	AT	*ATM*	Het Het	pathogenic pathogenic	NM_000051.4:c.450_453del NM_000051.4:c.5255_5256insCT	p.Ser151_Val152delinsTer p.Tyr1753PhefsTer4	0.000003098 -	Pathogenic (3) Pathogenic (1)	[38] -
P38	CHARGE (initial Unclassified)	*CHD7*	Het	pathogenic	NM_017780.4:c.4393C>T	p.Arg1465Ter	-	Pathogenic (6)	[39,40]
P51	Shwachman-Diamond syndrome (initial Unclassified)	*SBDS*	Het Het	pathogenic likely pathogenic	NM_016038.4:c.258+2T>C NM_016038.4:c.410T>G	- p.Met137Arg	0.003632 6.195 × 10^−7^	Pathogenic/Likely pathogenic (40) Uncertain significance (1) in ‘Inborn genetic diseases’	[41,42] [43]
P24 *, P25 *, P35	XLA	*BTK*	Hemi	likely pathogenic	NM_000061.3:c.1581_1584del	p.Cys527TrpfsTer2	-	Pathogenic (2)	[44,45]
P43	XLA	*BTK*	Hemi	likely pathogenic	NM_000061.3:c.389del	p.Asn130ThrfsTer2	-	Pathogenic (1)	[46]
P101	XLA	*BTK*	Hemi	likely pathogenic	NM_000061.3:c.1603G>T	p.Val535Phe	-	-	[47]
P75	XLA	*BTK*	Hemi	VUS	NM_000061.3:c.359T>C	p.Leu120Pro	-	Uncertain significance (1)	-
P45	BENTA-like	*CARD11*	Het	VUS	NM_032415.7:c.388T>G	p.Phe130Val	-	-	[48,49]
P127	HIgM	*CD40LG*	Hemi	VUS	NM_000074.3:c.716T>G	p.Val239Gly	-	-	-

* these patients are brothers. Note: IEI, inborn errors of immunity; X-CGD, X-linked chronic granulomatous disease; APECED, autoimmune polyendocrinopathy-candidiasis-ectodermal dystrophy; AT, ataxia-telangiectasia; CHARGE syndrome, coloboma, heart defects, atresia of the choanae, growth retardation, genital anomalies, and ear abnormalities syndrome; XLA, X-linked agammaglobulinemia; BENTA, B-cell expansion with NF-κB and T-cell anergy syndrome; HIgM, hyper-IgM syndrome; ACMG, American College of Medical Genetics and Genomics; HGVS, Human Genome Variation Society; AF, allele frequency; gnomAD, Genome Aggregation Database; ClinVar, ClinVar archive; Het, heterozygous; Hom, homozygous; Hemi, hemizygous; VUS, variant of uncertain significance.

**Table 3:** Genetic variants identified in patients with an initial CVID-like phenotype.

Patient	Diagnosis	Gene	GT	ACMG Classification	HGVS Transcript	HGVS Protein	gnomAD AF Total	ClinVar Classification (Submissions)	References	Disease (IUIS)	Inheritance (IUIS)	Category (IUIS)	OMIM (IUIS)
P4	NFKB1 deficiency	*NFKB1*	het	Likely pathogenic	NM_003998.4:c.1929_1932del	p.Leu645ThrfsTer8	-	-	-	NFKB1 deficiency	AD	PAD	164011
P74	NFKB1 deficiency	*NFKB1*	het	Likely pathogenic	NM_003998.4:c.961_971delinsTTAATATTATATTTTGTAATATATTAATA	p.Asp321_Ile324delinsLeuIleLeuTyrPheValIleTyrTer	-	-	-	NFKB1 deficiency	AD	PAD	164011
P6	CTLA4 deficiency	*CTLA4*	het	Likely pathogenic	NM_005214.5:c.565dup	p.Met189AsnfsTer24	-	-	-	CTLA4 deficiency (ALPSV)	AD	Immune Dysregulation	123890
P73	CTLA4 deficiency	*CTLA4*	het	Likely pathogenic	NM_005214.5:c.160G>T	p.Ala54Ser	-	-	[66]	CTLA4 deficiency (ALPSV)	AD	Immune Dysregulation	123890
P13	CVID	*SOCS1*	het	VUS	NM_003745.2:c.187G>C	p.Asp63His	-	-	-	SOCS1 deficiency	AD haploinsufficiency	Immune Dysregulation	619375
P84	CVID	*SH2D1A*	hem	VUS	NM_002351.5:c.269dup	p.Asp91ArgfsTer13	-	-	-	SH2D1A (SAP) deficiency (XLP1)	XL	Immune Dysregulation	300490
P172	CVID	*IKBKB*	het	VUS	NM_001556.3:c.68C>G	p.Thr23Arg	-	-	-	IKBKB deficiency; EDA-ID due to IKBKB GOF mutation	AR; AD GOF	CID; CID syndromes	615592; 618204
P22	CVID	*TNFRSF13B*	het	Risk variant *	NM_012452.3:c.310T>C	p.Cys104Arg	0.005441	Conflicting classifications of pathogenicity; risk factor Pathogenic (19); Likely pathogenic (14); Uncertain significance (1)	[20,65,67,68]	TACI deficiency	AD or AR	PAD	604907
P116	CVID	*TNFRSF13B*	het	Risk variant *	NM_012452.3:c.542C>A	p.Ala181Glu	0.005386	Conflicting classifications of pathogenicity Pathogenic (13); Likely pathogenic (10); Uncertain significance (1)	[20,65,67]	TACI deficiency	AD or AR	PAD	604907

* Risk variant, a genetic susceptibility factor. This designation is descriptive and does not represent an ACMG classification category. Note: GT, genotype; het, heterozygous; hem, hemizygous; ACMG, American College of Medical Genetics and Genomics; HGVS, Human Genome Variation Society; AF, allele frequency; gnomAD, Genome Aggregation Database; ClinVar, ClinVar archive; IUIS, International Union of Immunological Societies; OMIM, Online Mendelian Inheritance in Man; AD, autosomal dominant; AR, autosomal recessive; XL, X-linked; GOF, gain-of-function; VUS, variant of uncertain significance; PAD, predominantly antibody deficiencies; CID, combined immunodeficiencies; EDA-ID, ectodermal dysplasia with immunodeficiency; ALPSV, autoimmune lymphoproliferative syndrome type 5; XLP1, X-linked lymphoproliferative disease type 1; SAP, SLAM-associated protein; TACI, transmembrane activator and CAML interactor; CAML, calcium-modulating cyclophilin ligand.

## Data Availability

The original contributions presented in this study are included in the article. Further inquiries can be directed to the corresponding authors.

## Supplementary Materials

The following supporting information can be downloaded at: https://www.mdpi.com/article/10.3390/ijms27177923/s1.
