# Supplementary material for: Genetic Heterogeneity of Inborn Errors of Immunity Revealed by Whole-Genome Sequencing: Insights from a Russian Patient Cohort
Source: Int J Mol Sci. 2026 Sep 5;27(17):7923. doi: 10.3390/ijms27177923 (PMC13565858; doi:10.3390/ijms27177923)
Supplement: Supplementary file 1 [file ijms-27-07923-s001.zip › Supplementary Figures.pdf]

Supplementary Figures

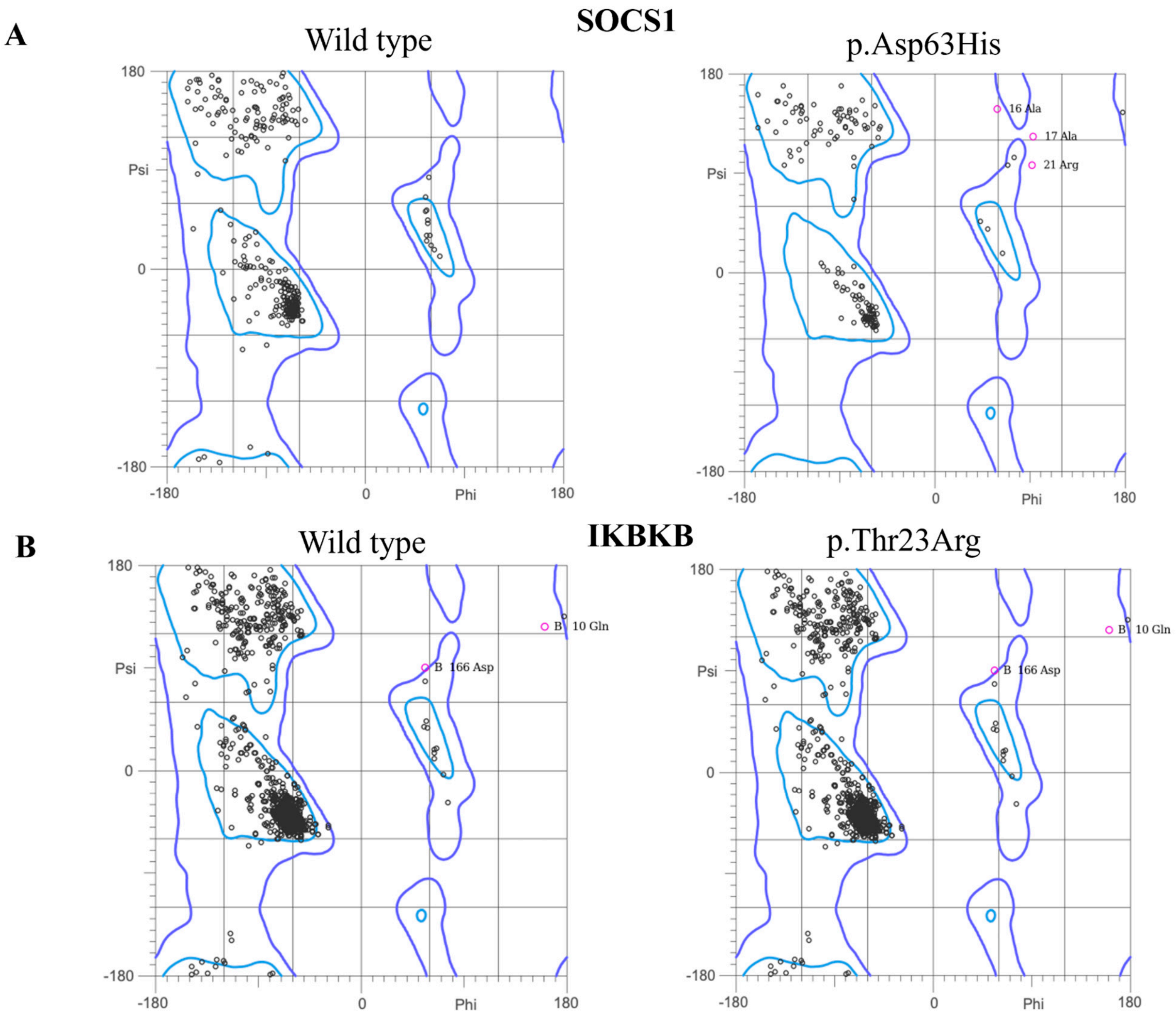

Supplementary Figure S1. Ramachandran plot analysis of wild-type and mutant SOCS1 and IKBKB structures. (**A**) Ramachandran plots for the wild-type SOCS1 structure and the mutant model carrying the p.Asp63His substitution. (**B**) Ramachandran plots for the wild-type IKBKB structure and the mutant model carrying the p.Thr23Arg substitution.

The plots display the distribution of φ and ψ dihedral angles for amino acid residues in the modeled proteins. The variants p.Asp63His in SOCS1 and p.Thr23Arg in IKBKB occur at highly conserved positions. Computational stability analysis using DynaMut2 did not reveal significant changes

in global protein stability for either variant: the SOCS1 p.Asp63His substitution showed a marginal predicted stabilizing effect ($\Delta\Delta G$ = +0.12 kcal/mol), while IKBKB p.Thr23Arg showed a marginal destabilizing effect ($\Delta\Delta G$ = −0.11 kcal/mol). Given that both values fall well within the error margin of the method (~1 kcal/mol), neither variant is predicted to cause substantial global structural disruption, which is consistent with the absence of major Ramachandran plot outliers in the mutant models. Notably, the SOCS1 stability analysis was performed on a structure in complex with JAK1 kinase; therefore, the predicted $\Delta\Delta G$ reflects the stability of the complex rather than isolated SOCS1. These data indicate that p.Asp63His does not cause substantial global thermodynamic destabilization of SOCS1. The affected residue, Asp63, is invariantly conserved across vertebrate orthologs and is precisely localized within the kinase inhibitory region (KIR) — the primary structural determinant of SOCS1-mediated JAK kinase suppression — suggesting that this position is functionally critical regardless of its effect on overall protein stability.

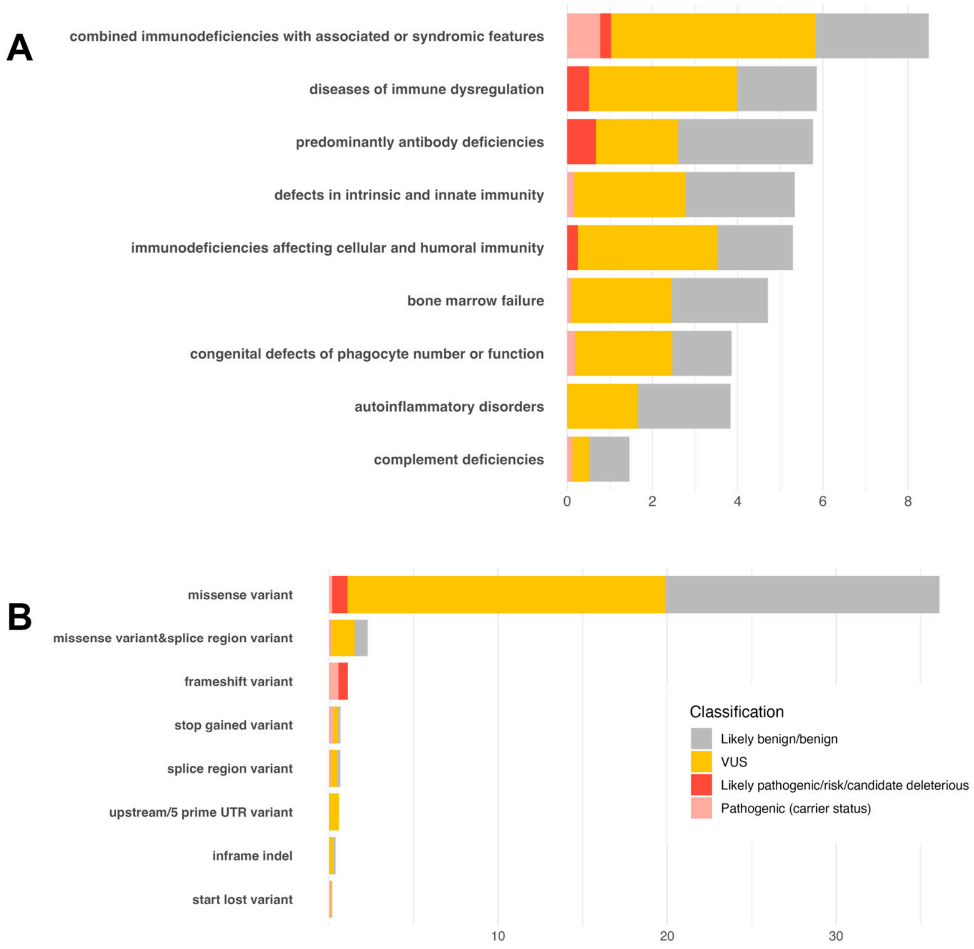

Supplementary Figure S2. Patient-normalized distribution of rare variants (MAF<0.005) in IEI genes. (a) the distribution of the weighted number of retained rare variants across IEI categories (defined by gene-disease association). (b) the same patient-normalized analysis for variant consequence categories (predicted impact on the protein/transcript).

To explore the genetic background of the CVID cohort, we focused on rare, pre-filtered variants and summarized their functional annotation profile at the patient level. To avoid over-weighting individuals with a large number of retained variants, we applied per-patient normalization: for each patient p with np retained rare variants, each variant was assigned a weight w=1/np, so that the total weight contributed by each patient equals 1. Weighted contributions were then aggregated across the cohort within each annotation category (IEI category or predicted consequence) and stratified by variant classification (likely benign, VUS, or likely pathogenic/risk/candidate deleterious), yielding an average per-patient distribution.

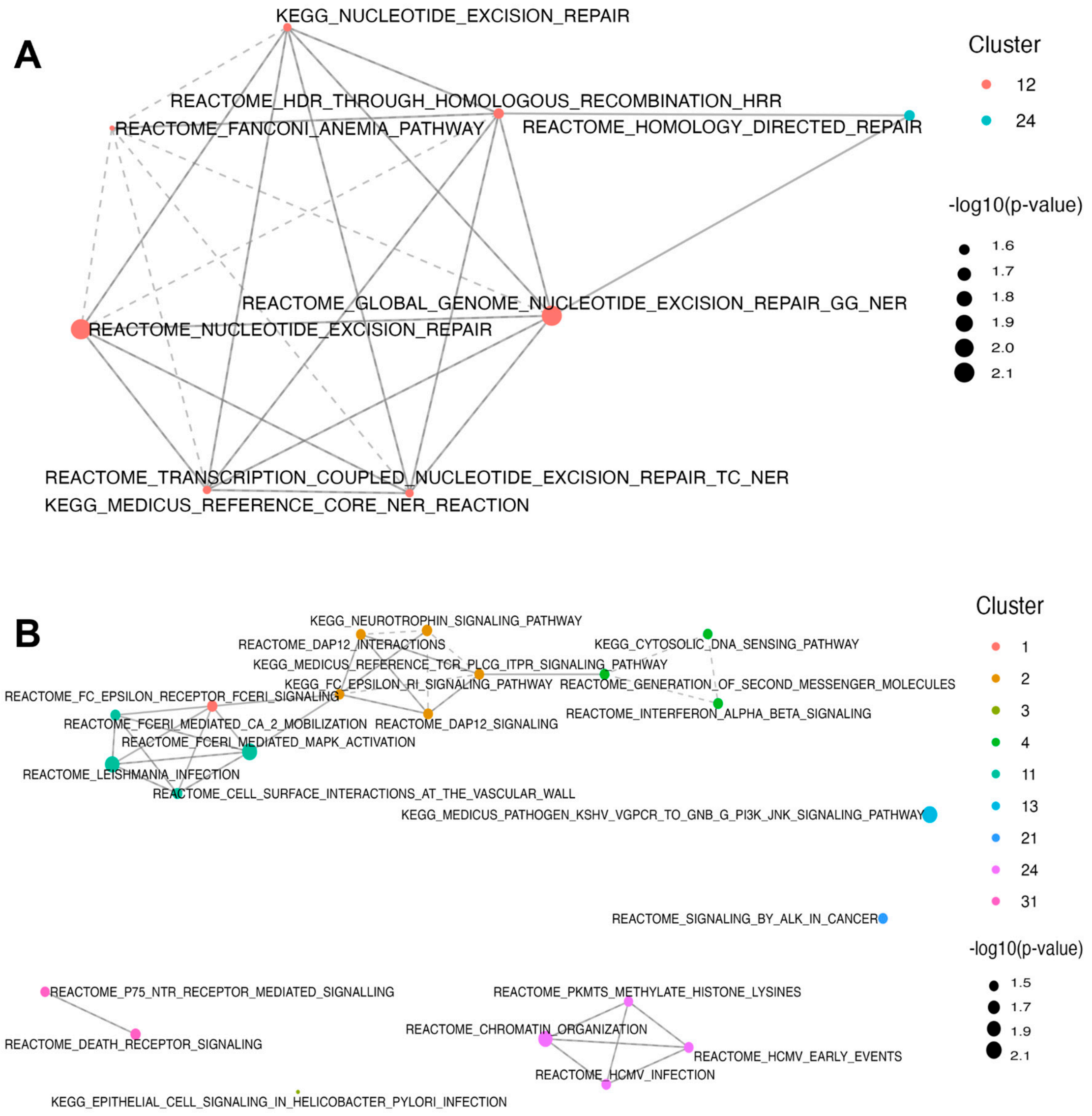

Supplementary Figure S3. (**A**) Network representation of enriched DNA repair pathways identified in patients with autoimmune manifestations. Each node represents a pathway, with node size proportional to pathway significance (−log10 p-value). Edges indicate gene overlap between pathways (Jaccard similarity > 0.15), illustrating the formation of a functionally related DNA repair cluster including nucleotide excision repair and homology-directed repair processes. (**B**) Network representation of significantly enriched pathways ($p < 0.05$) in patients with polyclonal lymphocytic infiltration. Each node represents a pathway, with node size proportional to pathway significance (−log10 p-value). Node colors indicate

pathway clusters identified by Louvain community detection based on pathway overlap (Jaccard similarity > 0.15). Solid edges denote direct gene overlap between pathways, while dashed edges indicate additional links between pathways belonging to the same cluster, highlighting the structure of functional immune signaling modules.

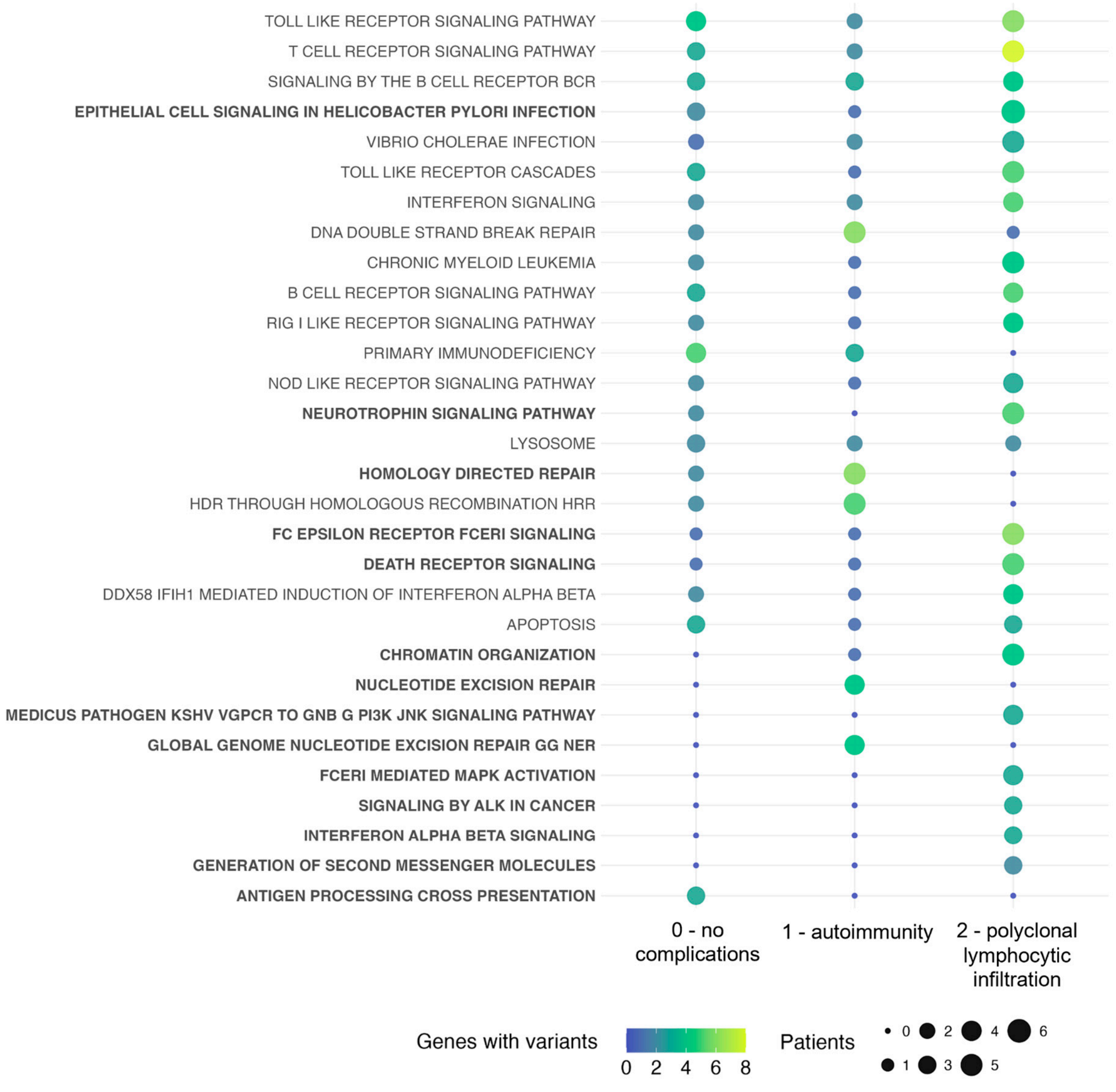

Supplementary Figure S4. Distribution of genetic variants across signaling pathways in clinical subgroups of CVID. Each bubble represents a pathway within a specific clinical category. Bubble size corresponds to the number of patients carrying variants in genes associated with the pathway, while color indicates the number of distinct genes affected. Pathways significantly enriched according to Fisher's exact test are shown in bold. Only Fisher‑significant pathways together with the most frequent additional pathways are displayed to highlight the main biological signals across clinical subgroups. Selected pathways included for visualization in this figure are listed in **Supplementary Table S9**.
